# Biology of *Eumacronychia* Townsend, with a redescription of *E.persolla* Reinhard, 1965 (Diptera, Sarcophagidae)

**DOI:** 10.3897/zookeys.783.28057

**Published:** 2018-09-03

**Authors:** Wen-tian Xu, Dong Zhang, Thomas Pape

**Affiliations:** 1 College of Nature Conservation, Beijing Forestry University, Beijing 100083, China Beijing Forestry University Beijing China; 2 Natural History Museum of Denmark, University of Copenhagen, Universitetsparken 15, DK–2100 Copenhagen, Denmark niversity of Copenhagen Copenhagen Denmark

**Keywords:** Miltogramminae, flesh flies, taxonomy, morphology, habitus, male terminalia

## Abstract

The biology of *Eumacronychia* Townsend is reviewed and *Eumacronychiapersolla* Reinhard, 1965 is redescribed. The male and female habitus as well as the male terminalia are documented with focus-stacked photographs, and features separating this species from all other species of *Eumacronychia* are discussed.

## Introduction

The genus *Eumacronychia* Townsend, together with *Euphyto* Townsend, *Gymnoprosopa* Townsend, *Gymnopsidia* Shewell and *Opsidia* Townsend, is one of only five genera of miltogrammine flesh flies endemic to the New World (Pape 1996, but see Shewell (1987) for a more fine-grained classification). In its current circumscription (Reinhard 1965, Shewell 1987, Pape 1996), *Eumacronychia* is morphologically very similar to the genus *Gymnoprosopa*, and these two genera appear to represent an ancient vicariance event, with the currently known 21 species of *Eumacronychia* largely confined to the western and southern parts of the Nearctic and the northern Neotropics, and species of *Gymnoprosopa* largely confined to the eastern Nearctic (Downes 1965, Pape 1996). *Eumacronychia* has been variously classified as belonging to the Miltogramminae (e.g., Downes 1965, Pape 1996, Szpila 2010) or the Paramacronychiinae (e.g., Verves 1990), or to the subfamily Eumacronychiinae composed of a mixture of miltogrammine and paramacronychiine genera (Verves 1998, 2001). Piwczyński et al. (2017) provided molecular data supporting a basal position of *Eumacronychia* in the subfamily Miltogramminae.

The male terminalia of species within the subfamily Miltogramminae are in general rather uniform, and species often show some degree of sexual dimorphism in their external morphology, e.g., males may have a silvery frontal vitta, a much stronger and more contrasting thoracic or abdominal colour pattern, structural modifications of legs like shortened or elongated tarsomeres and loss or elongation of claws, or modified chaetotaxy on the head, thorax, abdomen or legs. Species of the genus *Eumacronychia* differ from this pattern by showing little sexual dimorphism and a remarkable similarity in the adult habitus, but a considerable structural diversity in the male terminalia. This was noted by Reinhard (1965), who mentioned how species of *Eumacronychia* are “unusually homogeneous in external morphology so that almost without exception the members are not distinguishable from each other except by reference to the male terminalia”, and it is therefore surprising that he did not provide a single illustration and only described the male terminalia in simple and rather vague terms. Apart from this shortage, Reinhard’s (1965) key to species is well-structured and fully dichotomous except for entry 15, which contains a triplet separating *E.persolla* Reinhard and *E.duplicata* Reinhard as well as leading on to the remaining four species.

The aim of the present paper is to provide a review of what is known on the biology of *Eumacronychia* and to redescribe *E.persolla*, which is also a species with documented forensic importance (Szpila et al. 2010). We provide extensive documentation of the adults of both sexes of *E.persolla*, including the first photographs of the male terminalia, thereby providing improved means for its recognition.

### Biology of *Eumacronychia*

*Eumacronychia* is phylogenetically and biologically interesting as it has been shown to occupy a basal position in the subfamily Miltogramminae and to have retained a necrophagous breeding habit (Szpila et al. 2010, Piwczyński et al. 2017). Reinhard (1965) mentioned that “some species” of *Eumacronychia* had been collected from decomposing vertebrates and invertebrates on the beach and that adults could be seen to “move rapidly in flight over areas frequented by fossorial Hymenoptera”, while others had been collected feeding from plant nectaries. Apart from this, Reinhard (1965) claimed that “no information [is] available concerning the biology or host relationships of any member of [*Eumacronychia*]”. Reinhard evidently overlooked the information provided by La Rivers (1944) from a massive outbreak of Mormon crickets in 1939 in northern Nevada, where “two sarcophagid flies, *Eumacronychiaelita* Townsend and *Euarabatergata* (Coquillett) … invaded the burrows of *Chlorionlaeviventris* and utilised the paralyzed crickets as food for their own larvae and eggs. The sarcophagids, too small to dig, took advantage of the wasp’s absence from the burrow to larviposit up to 20 larvae on each cricket”. La Rivers (1944) provided no data on who did the identifications, and no mention was made of any voucher specimens, which means that these miltogrammine records cannot be confirmed.

Since Reinhard’s (1965) taxonomic revision, important biological data have been published. Cornaby (1974) provided a table where adults of *Eumacronychiasternalis* Allen were listed as collected from carrion of both toads and lizards in Costa Rica, but no evidence was given of larviposition or actual breeding. Pape and Dahlem (2010) also reported *E.sternalis* as being attracted to carrion, but gave no further details. Szpila et al. (2010) recorded repeated breeding in California of *E.persolla* from pig carcasses buried in pits of 0.40 m and 0.66 m depth, respectively, for forensic studies. A pig carcass from an open pit of 0.49 m depth was mainly colonised by *Chrysomyarufifacies* (Macquart), with the notable absence of *E.persolla* larvae. Valdés-Perezgasga et al. (2010) also recorded larvae of miltogrammine flesh flies on pig carrion in Mexico (Coahuilan semidesert) and identified the species as “*Anicia* sp.”. Szpila et al. (2015) corrected the identification to *Eumacronychia* sp. by checking digital photographs of adult flies bred from these larvae. The pig carcasses in the study of Valdés-Perezgasga et al. (2010) were deployed above-ground in a steel-rod cage covered with iron mesh. Similarly, Gautreau (2007) obtained larvae of *E.sternalis* from carrion traps (beef liver) buried randomly on a Costa Rican beach, with larvae reared successfully to adults. Unpublished notes from the late William Downes, Jr. (*in litteris* to TP 20 June 1995) report *E.agnella* Reinhard reared from hamburger meat in Sand Ridge State Forest, Illinois, USA, and an unidentified female of *Eumacronychia* bred from a dog carcass buried in a sand dune in California.

Mullen et al. (1984) and Trauth and Mullen (1990) described how larvae of *E.nigricornis* Allen live as predators on eggs of the iguanid lizard *Sceloporusundulatus* (Bosc & Daudin). *Eumacronychiasternalis* has been bred from eggs of leatherback, olive ridley and green sea turtles (Gautreau 2007, Lopes 1982), and similar breeding records of “*Phrosinella* sp.” and “*Eusenotainia* sp.” by López Barbosa (1989), Andrade et al. (1992) and Gámez-Vivaldo et al. (2006) are most likely misidentifications of *Eumacronychia* spp. Gautreau (2007) found larvae of *E.sternalis* to be capable of producing myiasis in turtle hatchlings (in both singular and mixed infestations), mostly in weakened individuals but in a few cases also in fully viable ones.

Evidently, species of *Eumacronychia* appear to be necrophagous, with the gravid female searching for vertebrate carrion buried at a modest depth in sandy areas. The larvae are deposited on the surface of the sand above the carrion and dig down to find their food. It appears that larvae of *Eumacronychia* can also act as predators of reptile eggs as well as weak hatchlings, which would be equal to grossly oversized prey.

## Materials and methods

Preparation and photography of male terminalia were done following the methods detailed in Zhang et al. (2013). Terminology follows Cumming and Wood (2018) for external morphology and Sinclair (2000) for male terminalia. High-resolution photographs of the holotype of *Eumacronychiapersolla* were examined to avoid the risks involved in shipping the specimen. Distributional data were taken from Pape (1996).

Abbreviations for depositories of material examined are as follows:

**CAS**California Academy of Sciences, San Francisco, USA.

**NHMD** Natural History Museum of Denmark, Copenhagen, Denmark.

## Taxonomy

### Eumacronychia persolla

Taxon classificationAnimaliaDipteraSarcophagidae

(Reinhard, 1965)

Figs 1 , 2 , 3 , 4

Eumacronychia persolla Reinhard, 1965: 349. Type locality: USA, California, Contra Costa County, Antioch. HT ♂, CAS.
E. personella : Lopes (1969: 3), incorrect subsequent spelling of persolla Reinhard, 1965.
E. persola : Lopes (1982: 427), incorrect subsequent spelling of persolla Reinhard, 1965.

#### Redescription.

**Figure 1.**
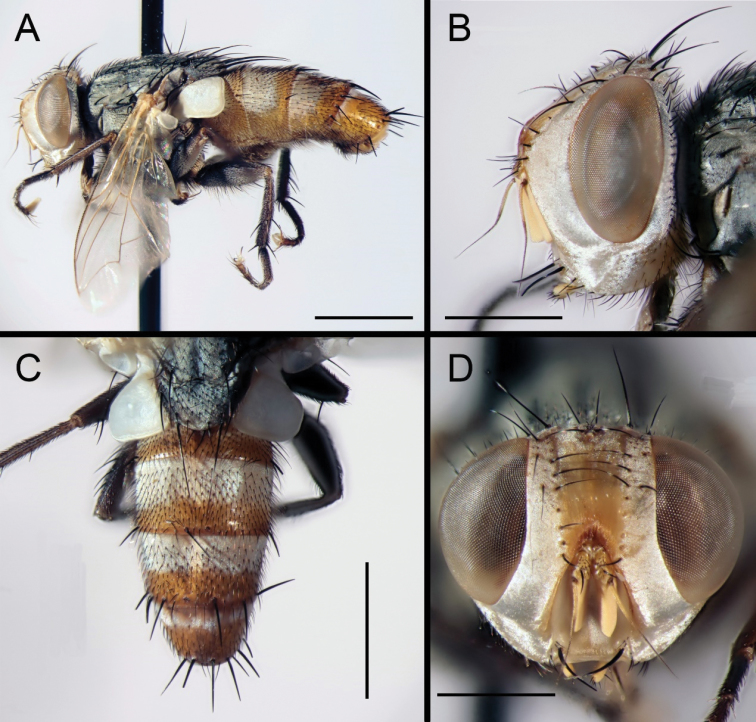
*Eumacronychiapersolla* Reinhard, 1965. ♂ from California, Imperial County, Algodones Dunes (NHMD). **A** Habitus, lateral view **B** Head, lateral view **C** Abdomen, dorsal view **D** Head, anterior view. Scale bars: **A, C** = 2.0 mm; **B, D** = 1.0 mm.

Male (Figs 1, 3, 4). Body length 8.0 mm (Fig. 1A). Head almost entirely yellowish, including gena, postgena, occipital bridge and median occipital sclerite. Occiput greyish black. Eyes bare. Fronto-orbital and parafacial plates with whitish or silvery grey pollinosity, parafacial about 1.5 × as broad as fronto-orbital plate at its narrowest point, bare or with some very small, black setulae forming an ill-defined brown colour antero-ventrally (i.e., along lower margin close to genal groove). Frontal vitta reddish yellow, wide, about 1.8 × as wide as fronto-orbital plate in median part, sparsely pollinose in upper part. Frons at vertex 0.3 × head width, with rows of 10 or 11 frontal setae. Outer vertical seta well differentiated from postocular setae. Inner vertical seta strong and more than 2.0 × as long as outer vertical seta. Two proclinate and one reclinate fronto-orbital setae. Fronto-orbital plate otherwise with only a few setulae at vertex. One pair of strong ocellar setae directed antero-laterally. Gena silvery pollinose on pale ground colour, with sparse and short black setae, 0.3 × eye height in lateral view. Antenna orange (postpedicel may be dirty orange). Arista entirely brown or with third aristomere orange in proximal part (up to 0.4 of length), 1.4 × as long as postpedicel, thickened in proximal half, with micropubescence (most distinct just distal to middle). Postpedicel about as long as palpus. Vibrissa well developed, with a few supravibrissal setulae. Proboscis brown. Palpus orange or dark yellowish.

Thorax black in ground colour; scutum with grey pollinosity and with three black dorsal vittae. Chaetotaxy: acrostichals 0+1, dorsocentrals 2+3, intra-alars 0, supra-alars 2, postalars 2, postpronotals 2, notopleurals 2; scutellum with two pairs of lateral, one pair of discal, and one pair of apical setae. Wing hyaline, subcostal sclerite and basicosta yellow; colour of tegula with at least the distal margin orangish or light brown; costal spine not differentiated; node of R_2+3_-R_4+5_ with 5–6 setae dorsally. Legs blackish. Fore femur with rows of postero-ventral and postero-dorsal setae; fore tibia with a row of six antero-dorsal setae and 1 sub-median posterior seta. Mid femur with 4 ventral setae and 2 posterior setae; mid tibia with a row of antero-dorsal setae, 2 postero-dorsal setae and 1 ventral seta. Hind femur with a row of 5 antero-dorsal setae and a row of 6 ventral setae in basal half; hind tibia with a row of antero-dorsal setae, 1 sub-median antero-ventral and 2 postero-dorsal setae.

Abdomen long oval, ground colour usually blackish except for tergite 5, which is reddish (dark or ‘dirty’ reddish anteriorly, although specimens with an almost entirely reddish abdomen are known (Fig. 1C), possibly a result of bleaching from storage in ethanol), tergites 3–5 each with a broad band of silvery pollinosity anteriorly, covering about 75% of tergite 3, 50% of tergite 4 and 30% of tergite 5. Tergite 3 with a pair of median marginal setae, tergites 4 and 5 with a complete row of strong marginal setae. Terminalia reddish to orange. Syntergosternite 7+8 bare. Epandrium with scattered short setae, especially in dorsal part. Cercus gently curved anteriorly in profile, tapering, basal third with long dense setae dorsally. Surstylus broad in proximal 0.8, narrowing to a truncated apex with postero-apical ‘corner’ slightly twisted laterally (Fig. 3B). Pregonite long and pointed; postgonite with a fine seta close to apex. Distiphallus elongate and sickle-shaped, with a small subapical constriction before the lightly sclerotised tip, ventro-median sclerotisation running the full length of distiphallus. Dorsal part of distiphallus entirely membranous, armed with small cuticular spines on most of its surface (Fig. 3A). Epiphallus short, tapering and slightly curved, with a pointed apex and slanting at an angle of about 45° (Fig. 3C).

**Figure 2.**
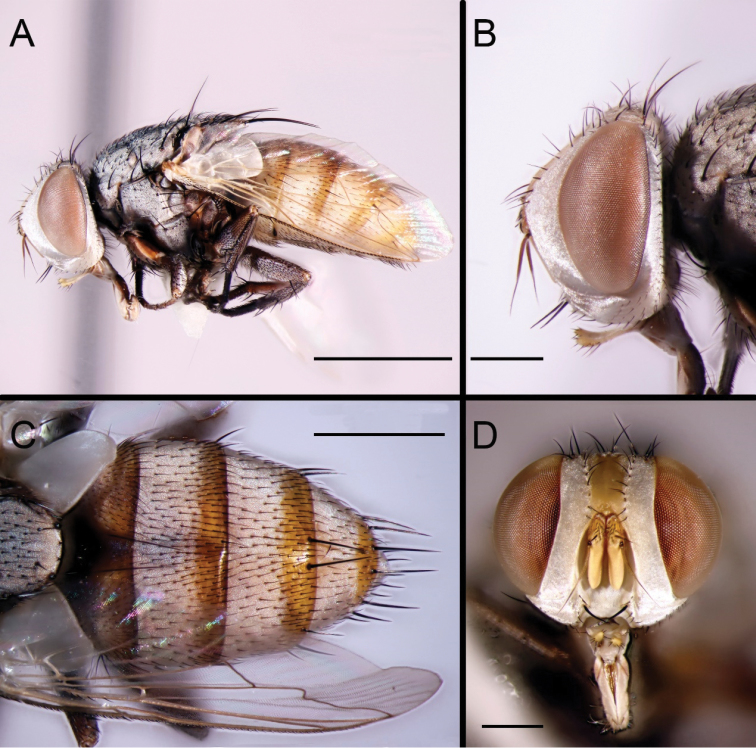
*Eumacronychiapersolla* Reinhard, 1965. ♀ from California, Imperial County, Algodones Dunes (NHMD). **A** Habitus, lateral view **B** Head, lateral view **C** Abdomen, dorsal view **D** Head, anterior view. Scale bars: **A** = 2.0 mm; **C** = 1.0 mm; **B, D** = 0.5 mm.

Female (Fig. 2). Body length 6.0 mm. Similar to male but with frontal vitta narrower and about 1.1 × as broad as fronto-orbital plate at its narrowest point. Abdomen broadly ovate, median marginal setae on tergite 3 absent.

#### Material examined.

Holotype male [examined from photographs]: USA, California, Contra Costa County, Antioch, 3.X. 1985, J Powell leg. (CAS); 1 male, 1 female: USA, California, Imperial County, Algodones Dunes, 33°02'N 115°08'W, 17–22.IX.2008, RB Kimsey & TJ Zavortink leg., Malaise trap (NHMD); 1 male: USA, California, Davis, 20.VII.1953, EC Carlson leg., “Fish meal bait” (NHMD); 1 male: USA, California, Sacramento, 31.VII.1952, PH Arnaud leg. (NHMD); 1 male: USA, Oregon, Josephine County, Whisky Creek, 27.VI.2002, W Reeves leg. (NHMD).

#### Remarks.

**Figure 3.**
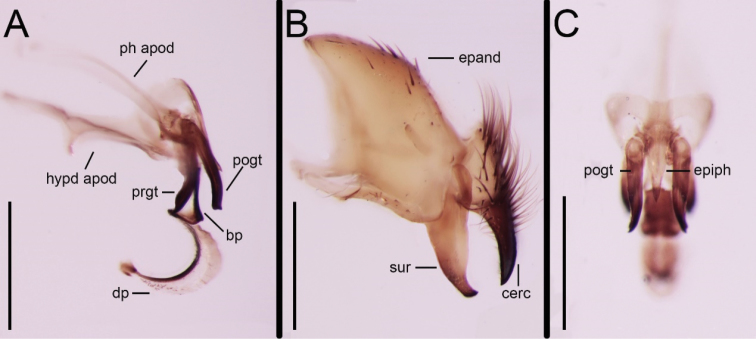
*Eumacronychiapersolla* Reinhard, 1965. ♂ from California, Imperial County, Algodones Dunes (NHMD). **A** Phallus and associated structures, lateral view **B** Epandrium, surstylus and cercus, lateral view **C** Phallus and associated structures, posterior view. Abbreviations: bp = basiphallus; cerc = cercus; dp = distiphallus; epand = epandrium; epiph = epiphallus; hypd apod = hypandrial apodeme; ph apod = phallapodeme; pogt = postgonite; prgt = pregonite; sur = surstylus. Scale bars: 0.5 mm.

**Figure 4.**
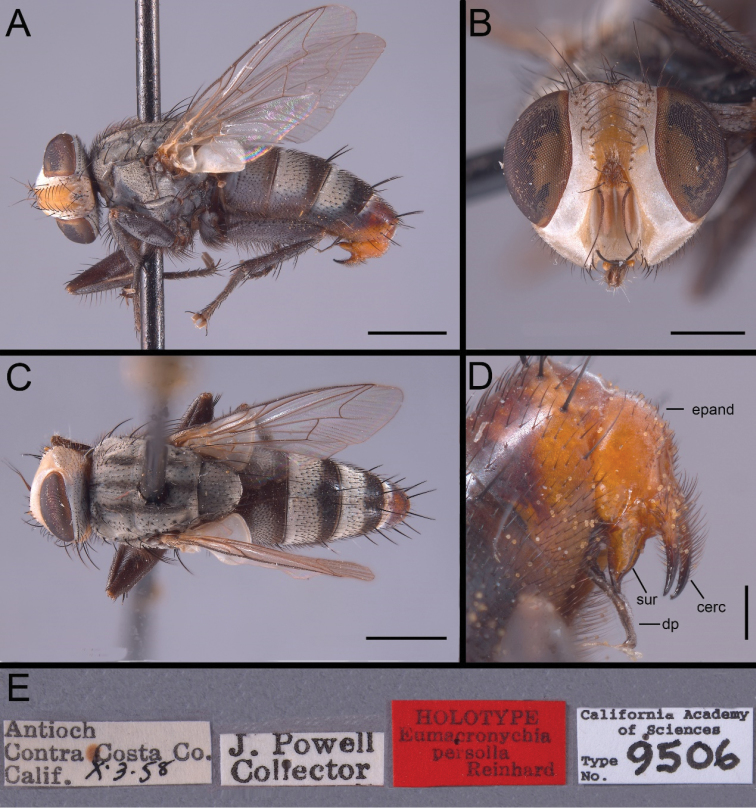
*Eumacronychiapersolla* Reinhard, 1965. Holotype ♂ from California, Contra Costa County, Antioch (CAS). **A** Habitus, lateral view **B** Head, anterior view **C** Habitus, dorsal view **D** Terminalia, lateral view **E** Labels. Abbreviations: cerc = cercus; dp = distiphallus; epand = epandrium; sur = surstylus. Scale bars: **A, C** = 1.0 mm; **B** = 0.5 mm; **D** = 0.2 mm. [California Academy of Sciences, photos by Rachel Diaz-Bastin]

The almost entirely reddish abdomen in both males and females from the Algodones Dunes (Figs 1, 2) is noteworthy, but the available material is insufficient to decide whether this is a natural condition or an artifact from being preserved in ethanol. Differences in the male terminalia between the holotype (Fig. 4D) and the male from Algodones Dunes (Fig. 3) are here considered to be due to differences in preservation as well as minor differences in orientation. The distiphallus of the dry-mounted holotype has the membranous lateral and dorsal parts of the distiphallus mostly collapsed against the ventro-median sclerotisation, while the KOH-treated phallus stored in glycerine and examined for the present study has the distiphallic membrane fully expanded (Fig. 3A). The slightly skewed cerci in Fig. 4D make them appear more slender than in the perfectly aligned and strict lateral view (Fig. 3B).

#### Distribution.

Nearctic – Mexico (Baja California Norte, Baja California Sur, Sonora), USA (Arizona, California, Idaho, Nevada, Oregon [new record], Utah, Washington).

## Discussion

Comparative studies of the male terminalia of *Eumacronychia* spp. are beyond the scope of the present paper, and the only figured male terminalia of species of this genus are those of *E.persolla* by Verves (1990) and of *E.sternalis* by Lopes (1982) and Gautreau (2007). Reinhard (1965) stressed the strap-like and strikingly elongate distiphallus of *E.persolla*, which would indicate that he had not observed any similar phallic configuration during his revisionary work. This is in agreement with observations made so far by one of us (TP). One feature that appears to readily separate *E.persolla* from all congeners without preparations of terminalia, and is equally effective for both sexes, is the colour of the tegula (WL Downes, pers. comm. to TP). In the material of *E.persolla* examined here, the colour of the tegula always has at least the distal margin orangish or light brown, while in all other species of *Eumacronychia* examined the tegula is entirely black.

## Supplementary Material

XML Treatment for Eumacronychia persolla
